# Change in Dental Visits Among Eligible Children Under the Impact of the Child Dental Benefits Schedule in Australia

**DOI:** 10.1111/cdoe.13036

**Published:** 2025-03-13

**Authors:** Lan Nguyen, Luke B. Connelly, Stephen Birch, Ha Trong Nguyen

**Affiliations:** ^1^ Centre for the Business and Economics of Health, the University of Queensland, Brisbane Brisbane Australia; ^2^ Department of Sociology and Business Law The University of Bologna Bologna Italy; ^3^ Centre for Health Economics, University of Manchester Manchester UK; ^4^ Centre for Health Economics and Policy Analysis, McMaster University Hamilton Canada; ^5^ The Kids Research Institute Australia Perth Australia; ^6^ Centre for Child Health Research, the University of Western Australia Perth Australia

**Keywords:** adolescents, children, dental care, government programs, longitudinal studies

## Abstract

**Objectives:**

In Australia, although there have been some improvements, child oral health continues to be a major public health issue. The Australian Government introduced the means‐tested Child Dental Benefits Schedule (CDBS) in 2014 to support access to dental services for children and adolescents aged 0–17 years from low‐income families. There is a lack of evidence documenting whether the CDBS improved the dental attendance rate. This study aimed to evaluate the impact of the CDBS on dental visits among eligible children and adolescents in Australia.

**Methods:**

The study analysed the data set from the birth cohort (B cohort) in the Longitudinal Study of Australian Children (LSAC). This is a nationally representative cohort survey collected biennially since 2004. The information on dental visits in the last 12 months was reported by the parents. A difference‐in‐differences analysis was used to examine 22,985 observations in the period 2008–2018. A propensity score matching (PSM) method was employed as a robustness check for the main findings.

**Results:**

The proportion of children and adolescents eligible for CDBS in the six biennial surveys from 2008 to 2018 was 62.0%, 54.4%, 47%, 41.2%, 35.5%, and 28.9%, while the proportion of eligible individuals visiting dentists was 38.0%, 45.6%, 53.0%, 58.8%, 64.5%, and 71.1%, respectively. The analyses showed that the CDBS policy had a statistically significant and positive impact on dental visits among eligible children and adolescents. There was a 6.1–6.4 percentage point increase (*p*‐value < 0.001) in dental visits across different specifications after the introduction of the CDBS policy.

**Conclusion:**

The removal of financial barriers was beneficial to improve dental visits; however, the target group still faces the other remaining barriers, especially those related to inequalities in the social determinants of health, impeding the uptake of free dental services.

## 1 Introduction

In Australia, despite some improvements, child oral health remains a significant population health problem in the 21st century, with more than 30% of children aged 5–6 years and nearly 40% of children aged 12–14 years experiencing dental caries in the deciduous and permanent dentition, respectively [1]. Dental caries is one of the leading causes of the burden of disease among children aged 5–14, following asthma and mental health disorders [2].

To support access to dental services for children and adolescents from low‐income families and to limit the impact of children's poor oral health, the Australian Government introduced a means‐tested dental care policy named Child Dental Benefits Schedule (CDBS) in 2014 to cover part or all the cost of some dental services for eligible children aged 0–17 years (aged 2–17 years before the year 2022). The CDBS provides up to $1,052 in benefits (the benefit cap, indexed annually) over a relevant two‐calendar‐year period for dental services in a public or private setting, including examinations, X‐rays, cleaning, fissure sealing, fillings, root canals, extractions, and partial dentures; but the benefits are not available for orthodontic, cosmetic dental work, or any services provided in a hospital. A child is eligible if their family receives a relevant Australian Government payment at any point in the calendar year. Most eligible children satisfy the means test because their family receives Family Tax Benefit Part A. Services Australia assesses the eligibility and sends out a notification letter to eligible families. More details of the CDBS and its utilisation rates over the years can be found in the report on the fifth review of the Dental Benefits Act 2008 [3].

Before the introduction of the CDBS, the Australian Government implemented the Chronic Disease Dental Scheme (CDDS) from 2007 to 2012 and the Medicare Teen Dental Plan (MTDP) from 2008 to 2013 to improve access to dental services and address dental health inequalities. The two programs were evaluated as not effective and poorly utilised, especially in rural and remote areas ([4, 5]; Australian National Audit Office [6]; Department of Health [7]).

Like the previous programmes, the utilisation rate of the CDBS was considerably lower than originally projected [3], although there was a significant increase in the scope of dental services to cover basic dental needs and an increased age range of eligible children compared to the MTDP. From 2014 to 2021, the utilisation rates have increased slightly (except for 2020 due to the impact of the COVID‐19 pandemic) but in general, utilisation represents just over one‐third of the eligible population [3].

To date, several studies have examined reasons why the utilisation of the CDBS was lower than anticipated (e.g., [8, 9, 10, 11]). There is, however, little research on whether the CDBS improved the dental attendance rate among eligible children. Stormon et al. [12] studied the impact of CDBS on dental visits by using data from both cohorts K and B from the Longitudinal Study of Australian Children (LSAC) in the Poisson models. The LSAC is an ongoing, biennial national survey that started in 2004 and consists of the birth cohort (cohort B, aged 0–1 years at wave 1) and the kindergarten cohort (cohort K, aged 4–5 years at wave 1). For more details on this dataset, refer to Section 2.

It would appear difficult to disentangle the impact of the MTDP and the CDBS on dental visits because cohort K in the survey 2014 can use either the MTDP (which was designed for eligible teenagers aged 12–17 and closed at the end of the year 2013) or the CDBS to visit dentists (with parents reporting dental visits in the last 12 months in the survey).

This study aimed to identify separately the effect of the ongoing CDBS policy on the dental attendance rate among eligible children. Panel data methods, clustered at the individual level, were used to address the potential impact of time‐invariant unobservable factors and serial correlation in the dataset, which could lead to biased results in a longitudinal regression model. Comprehensive explanators of dental visit demand were also introduced in the model to mitigate the impact of omitted variable bias in the existing literature. Finally, a variety of propensity score matching methods were conducted as robustness checks to assess the veracity of the results obtained from other regression models.

## 2 Methods

A difference‐in‐differences (DiD) generalised linear regression was used to assess changes in dental visits before and after the implementation of CDBS, an approach employed in previous studies to evaluate the effect of public policy on dental care in other countries [13, 14, 15, 16]. To account for possible serial correlations, all analyses used the robust standard errors clustered at the individual level at which treatment is independently assigned [17]. Stata/MP 17.0 (Stata Corp LP 2022) was used for the analyses.

Among theoretical frameworks developed in the literature to support the studies of healthcare access, Andersen's behavioural model of health service use is the most commonly employed framework. Healthcare utilisation was explained by the combination of three core factors: predisposing, enabling, and need factors, which all determine health behaviour [18].

It is hypothesised that the CDBS improves dental visits among eligible children as it removes financial barriers and represents an enabling factor in Andersen's behavioural model. The eligibility of children to the CDBS was identified by whether parents receive the family tax benefits or the parenting payments, similar to the previous study which identified the eligible children using the LSAC data [9, 12].

The treatment and control groups in the DiD design are eligible and non‐eligible children for the CDBS policy, respectively. The estimated equation is:

(1) $$Y_{\mathrm{it}}=\beta_{0}+\beta_{1}{\text{Post}}_{t}+\beta_{2}{\text{Treat}}_{i}+\beta_{3}\;{\text{Post}}_{t}*{\text{Treat}}_{i}+X_{\mathrm{it}}+\varepsilon_{\mathrm{it}}$$

The study outcome *Y*_*it*_ is an indicator of whether the individual had a dental visit in the last 12 months. *Post*_*t*_ is an indicator that equals 1 if the observation year is after 2014 (the implementation year of the CDBS policy) and zero otherwise. *β*_1_ represents the aggregate effect of factors on dental visits over time other than the effects of the CDBS programme. *Treat*_*i*_ is an indicator that equals 1 if the individual is eligible for the CDBS and zero otherwise. *β*_2_ represents the average difference between the treatment and control groups in dental visits before the introduction of the policy. The coefficient of interest is the interaction term (*β*_3_) representing the effects of the CDBS policy on dental access.

Included in *X* are the characteristics of children, children's families, and the socio‐economic index for areas (SEIFA) which the Australian Bureau of Statistics (ABS) has developed. The ancillary private health insurance variable was expected to be included in the model as it provides dental access for the insurance holders. However, the LSAC does not provide information about the ancillary cover. Nevertheless, family income is likely correlated to the probability of holding this voluntary insurance. ε denotes the error term. The indexes *i* and *t* represent individual and time, respectively.

To conduct the robustness check for the regression‐based approach, we employed a propensity score matching (PSM) method. The two methods are complementary and are most effective when used in combination [19]. First, the common support region was assessed by a logit regression for the probability of the treatment assignment (eligibility of the CDBS policy) to estimate the propensity scores. In the logit model, the characteristics of the child, the child's family, and SEIFA hypothesised to be related to both the treatment assignment (i.e., eligible to the CDBS) and the outcome of interest (i.e., dental visits) were included as the baseline characteristics [19, 20, 21]. Based on these scores, the overlap region for the probability of being treated and untreated was identified. After identifying the overlap region, the propensity scores were matched within the region of common support.

To check the validity of the matching, the covariate balance between the treated and untreated groups and the variance ratios was tested. The similarity of the baseline characteristics in the matched sample was compared by standardised mean differences [20]. In addition, the ratio of variances of the propensity score and covariates from the treated and untreated groups should be near one if the two groups are balanced [22]. This is, indeed, the case with our results (see Tables A5 and A6 in the Appendix 1).

Data were obtained from the B cohort in the LSAC. The LSAC is a nationally representative cohort survey collected biennially since 2004 and provides many aspects of children's developmental outcomes and their family, community, and society characteristics. At the baseline, the B cohort (birth cohort) was aged 0–1 years (5,107 infants). The LSAC's population weights were used to account for the complex survey design and to reflect the population level. Further information on the LSAC and the survey design is available from the LSAC's documentation and technical papers (Australian Institute of Family Studies [23]). Responses were collected over six consecutive surveys from the B cohort in 2008–2018, and the final sample has 22,985 observations.

This sample study rules out potential bias from previous dental policies (i.e., the CDDS and the MTDP) because children in the B cohort were not eligible for those policies. The CDDS, which ended in 2012 while these children were 8–9 years old, was aimed at supporting older people suffering from chronic diseases. The MTDP, which ended in 2013, did not apply to children in the B cohort, as they were 9–10 years old in 2013, while the program was designed for teenagers aged 12–17.

## 3 Results

Table 1 presents the characteristics of the overall sample on average from 2008 to 2018, as well as the characteristics of the eligible and non‐eligible children's samples.

**TABLE 1:** Sample characteristics over 2008–2018.

Variables	Overall sample, *N* = 22 985, (%, 95% CI)	Eligible children, *N* = 9672, (%, 95% CI)	Non‐eligible children, *N* = 13 313, (%, 95% CI)
Child characteristics			
Gender (Male)	51.2 (50.6–51.9)	52.5 (51.5–53.6)	50.2 (49.3–51.1)
Indigenous status (No)	96.6 (96.3–96.8)	94.7 (94.2–95.1)	98.0 (97.7–98.2)
Age in months (mean, 95% CI)	113.2 (112.7–113.8)	102.4 (101.6–103.2)	121.6 (120.8–122.3)
Brushing teeth at least 2 times/day	70.0 (69.3–70.6)	67.7 (66.7–68.6)	72.7 (70.9–72.5)
Ever had dental problems (No)	49.2 (48.5–49.9)	52.0 (51.0–53.1)	47.0 (46.2–47.9)
Family characteristics			
Mother's education (At least bachelor's degree)	56.3 (55.6–57.0)	48.7 (47.6–49.7)	62.1 (61.2–63.0)
Mother's employment (Yes)	73.2 (72.5–73.8)	57.9 (56.9–59.0)	84.9 (84.2–85.5)
Mother's health (excellent/very good/good)	91.1 (90.6–91.4)	88.1 (87.4–88.8)	93.3 (92.8–93.7)
Mother's smoking (No)	83.4 (82.9–83.9)	75.9 (75.0–76.8)	89.2 (88.6–90.0)
Mother speaking English at home (Yes)	85.7 (85.2–86.2)	86.1 (85.3–86.9)	85.4 (84.7–86.0)
Homeowner (Yes)	74.7 (74.1–75.3)	60.5 (59.4–61.5)	85.7 (85.0–86.3)
Biological parent co‐habits (Yes)	78.7 (78.1–79.3)	66.7 (65.7–67.7)	88.0 (87.4–88.6)
Number of siblings in house (mean, 95%)	1.5 (1.5–1.5)	1.7 (1.7–1.8)	1.35 (1.33–1.37)
Family's income			
First quartile (highest)	25.7 (25.1–26.3)	6.4 (5.9–6.9)	40.6 (39.7–41.5)
Second quartile	24.4 (23.8–25.0)	20.3 (19.5–21.2)	27.5 (26.7–28.3)
Third quartile	23.8 (23.2–24.4)	34.9 (33.9–35.9)	15.2 (14.6–15.9)
Last quartile (lowest)	26.2 (25.6–26.8)	38.4 (37.4–39.5)	16.7 (16.0–17.4)
Residential Characteristics (SEIFA)			
First quartile (Most advantaged area)	25.0 (24.4–25.5)	14.6 (13.8–15.3)	33.0 (32.1–33.8)
Second quartile	24.7 (24.1–25.0)	22.3 (21.4–23.2)	26.6 (25.8–27.4)
Third quartile	24.9 (24.4–25.5)	27.9 (27.0–28.9)	22.6 (21.9–23.3)
Last quartile (Least advantaged area)	25.4 (24.8–26.0)	35.2 (34.2–36.2)	17.8 (17.2–18.5)

*Note:* All figures were population‐weighted.

Abbreviation: 95% CI, 95% confidence interval.

The sample included slightly more boys (51.2%) than girls. Nearly 60% of participants brushed their teeth at least 2 times per day and the majority have non‐Indigenous status. The proportions of family income in the different quartiles were largely similar. Mothers obtaining at least a degree of bachelor level accounted for 56.3%, with 73.2% being employed (full‐time or part‐time). Most mothers spoke English at home (85.7%), reported their health as excellent/very good or good (91.1%), and did not smoke cigarettes. For the neighbourhood status, the proportions of families living in the different area‐based advantages were similar.

**FIGURE 1:**
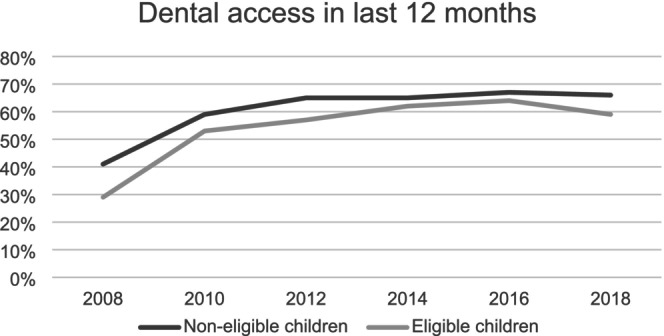
Pre‐trends in access to dental care in the last 12 months. Authors' illustration based on data collected from 2008 to 2018 of the LSACsurvey relating to the dental visits in the last 12 months of non‐eligible and eligible children.

Table 2 reports the trends in dental visits in the last 12 months in each year for two groups (eligible vs. non‐eligible). The trend of visiting dentists increases with age for both groups. First, the parallel trend assumption was assessed in dental visits between eligible and non‐eligible children in the pre‐reform period. As presented in Figure 1, the graphs did not show any significant divergence in the trends of dental visits between the two groups before 2014. Overall, the pre‐trends were similar (In addition, a placebo implementation year was conducted, and the results supported the DiD design, see Table A1 in the Appendix 1).

**TABLE 2:** The proportion of dental visits in the last 12 months before and after the introduction of the Child Dental Benefits Schedule.

Year (age)	Observations (individuals)	Eligible children (%)	Eligible children visiting dentist (%)	Non‐eligible children (%)	Non‐eligible children visiting dentist (%)
2008 (4–5)	4386	62.0	29.4	38.0	40.6
2010 (6–7)	4242	54.4	53.2	45.6	59.1
2012 (8–9)	4085	47.0	57.4	53.0	65.1
2014 (10–11)	3764	41.2	61.9	58.8	65.3
2016 (12–13)	3381	35.5	63.5	64.5	67.1
2018 (14–15)	3127	28.9	58.8	71.1	65.5

The main results of the CDBS policy's impact on dental visits obtained in the regressions are reported in Table 3. The results showed that the CDBS policy has a statistically significant impact at the 1% level on dental visits among eligible children and the impact remained stable across different specifications.

**TABLE 3:** Results of the Difference‐in‐differences regressions.

Variable	Fixed‐effects panel data analysis		Random effects panel data analysis	Pooled data analysis
	Coeff. model 1	Coeff. model 2	Coeff. model 3	Coeff. model 4
Post (*β*_1_)	0.094*** (0.009)	−0.099*** (0.013)	−0.111*** (0.012)	−0.113*** (0.013)
Treat (*β*_2_)	−0.029** (0.012)	−0.009 (0.011)	−0.024** (0.010)	−0.024** (0.010)
**DiD estimate (*β***_**3**_ **)**	0.063*** (0.015)	0.064*** (0.015)	0.063*** (0.013)	0.061*** (0.014)
Control variables	No	Yes	Yes	Yes
Observation	22 985	22 984	22 984	22 984

*Note:* **, ***‐Statistically significant at 5% and 1% level, respectively. Robust standard errors are in the brackets. Fixed‐effect panel data and pooled data regressions were weighted. Standard errors were clustered at the individual level.

In the model (2) adjusted for individual control variables, the estimated marginal effect (dy/dx) of the CDBS policy on dental visits is 0.064 (*p*‐value < 0.001) meaning that there was a 6.4 percentage point increase (95% CI: 3.6%–9.3%) in dental visits after the introduction of the policy (approximately equivalent to 103 eligible individuals in the sample). A related study showed that the introduction of the CDBS increased the rate of dental attendance for the group (low‐income) by 8% (95% CI: 1%–15%) [12]. By estimating the impact of the CDBS on dental visits for eligible individuals, a narrower range of confidence intervals was obtained.

Sensitivity analysis was performed by using an alternative sample that excluded observations from 2014 to avoid potential pre‐introduction measurement of dental visits in that year. The impact of CDBS on dental visits in this analysis was similar to the main findings (see Table A3, Appendix 1).

Possible attrition bias in a panel data analysis was expected to be adjusted by combining an attrition function in the main DiD model [24]. However, it was difficult to identify a variable in the survey that predicted the dropouts but did not directly influence the outcome of interest (dental visits). Nevertheless, the attrition rates were not high (smaller than 10%, except for the year 2016) and the presence of attrition does not necessarily generate biased estimates [25]. On the other hand, a longitudinal data setting is also useful for an analysis of policy impact [26]. Model (4) presents the result of pooled cross‐sectional data over time, and the marginal effect of the CDBS policy was 0.061 (*p*‐value < 0.001), very close to the estimates in the panel data analysis. Therefore, it may suggest that attrition did not appear to have a significant influence on the inference.

The findings showed statistically significant associations between dental visits and variations in the predisposing and need factors (see Table A2 in the Appendix 1). Regarding the need indicator, the coefficient on dental visits of children who had dental problems was positive and statistically significant at the 1% level. Children with dental problems were approximately 30 percentage points (*p*‐value < 0.001) (model 2) more likely to have dental visits within 12 months than children without dental problems. The finding reflects that a greater need for dental services was among the groups with dental problems. For the sensitivity test, the variable reflecting dental problems in the last 2 years was used in the model instead of the cumulative variable “ever had dental problems”, and similar findings were obtained.

Regarding the predisposing factors, the findings demonstrated the persistence of oral health inequalities among disadvantaged groups. Children associated with greater dental care usage were those from non‐Indigenous status, English‐speaking backgrounds, higher household income, employed mothers, and non‐smoking mothers (a proxy of health literacy). The findings suggested the significant impact of residential areas on dental attendance. For example, children living in the least advantaged area were 4.8 percentage points (*p*‐value < 0.05) (model 2) less likely to have a dental visit than those living in the most advantaged area. Dental practices have been distributed unevenly across Australia by socioeconomically disadvantaged areas and geographic remoteness [27].

**FIGURE A1:**
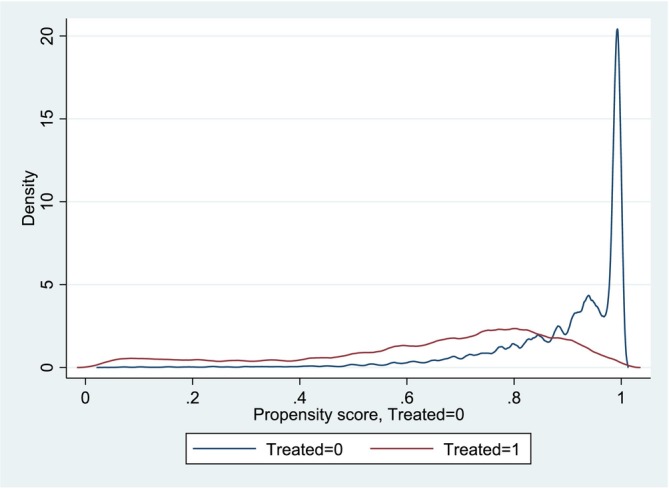
Distribution of propensity scores of being eligible and non‐eligible for the Child Dental Benefits Schedule in the region of common support.

PSM methods were employed in the dataset as a robustness check on the results obtained from the DiD generalised linear regression models. First, the common support region was identified, and observations in the unsupported region were dropped. The final sample consisted of 3184 treated and 19 539 untreated observations (see Table A4 and Figure A1 in the Appendix 1).

There are several variations of matching methods. For comparison, the average treatment effects of the CDBS policy on dental visits were estimated by different matching methods, including PSM, using a calliper width of 0.2 of the standard deviation, nearest‐neighbour matching (NNM) and inverse probability weighting (IPW). Table 4 presents the matching results.

**TABLE 4:** Average treatment effects of the child dental benefits schedule on dental visits.

Treatment	PSM Coeff. (Robust SE)	NNM Coeff. (Robust SE)	IPW Coeff. (Robust SE)
Being eligible for CDBS (ATT)	0.066*** (0.015)	0.067*** (0.014)	0.064*** (0.016)
All observations	22 723	22 723	22 723
Matched observations	6, 368	6, 368	—
Weighted observations	—		22, 723

*Note:* ***‐ Statistically significant level at 1%. 95% CI from the PSM, NNM, and IPW methods are 3.8–9.4, 4.0–9.5, and 3.3–9.4, respectively.

Abbreviation: ATT, Average treatment on the treated.

Table 4 shows the estimates of the policy effect remained relatively stable across these estimators and provided the same finding. All indicators suggested that there was an increase in dental visits among the treated group than the untreated group, highlighted by the significance of coefficients in all matching methods at the 1% level. The ATT was 6.4%–6.7%, implying there was a 6.4–6.7 percentage point increase in dental visits due to the impact of the CDBS policy. These estimates were very close to the estimates from the regressions in Table 3.

The validity of the matching was confirmed as the balance tests showed that PSM, NNM, and IPW matching methods achieved a sufficient balance of covariates between the comparable groups. All standardised mean differences were smaller than 25%, and the variance ratios in these methods were all near one [22, 28] (see Tables A5 and A6 in the Appendix 1).

## 4 Discussion

This study explored the impact of the ongoing CDBS on dental attendance among eligible children. The result was statistically significant, although the practical impact appeared to be quite modest, with a 6.4–6.7 percentage point increase in dental visits among eligible children due to the introduction of the CDBS. Relevant studies in other countries have also reported a similar effect size of dental programmes on low‐income residents [16, 29]. The small positive effects on the utilisation of the CDBS we observe highlight the fact that simply expanding access to dental services may not be enough to satisfy a policy goal of achieving greater service use by target groups.

Using rigorous statistical models, this study provides additional evidence of the factors influencing the effectiveness of the dental care policy in Australia. These aligned results may be conceived in terms similar to those put forward in Andersen's behavioural model, particularly the impact of predisposing factors and other enabling factors, such as ethnicity, socio‐economic status, health literacy, and the healthcare environment.

To improve access to dental care and the uptake of the CDBS benefits, more effort is needed to address the barriers regarding inequalities in the social determinants of health through collaboration with other government departments. Baum et al. [30] noted that, in Australia, there are few instances of cross‐sectoral action from the health sector that are aimed at addressing and modifying the social determinants of health that sit outside the health sector. Indeed, health policies often focus on increases in average health status rather than reducing inequities [31].

Since dental caries remain the most prevalent burden‐of‐disease category for children aged 5–14 in Australia, the CDBS is an important initiative to support access to dental care for vulnerable children. As was observed by Nguyen et al. [9], non‐financial considerations may be important to address if greater coverage of the eligible population is a goal of national policy in this area.

There are several limitations of the work reported in this paper. First, the study relied on dental visit information that was self‐reported and, as such, is likely to be subject to recall bias. Additionally, using cohort data, the results might be affected by the cohort effect and may not present all age groups eligible for the CDBS. Despite its limitations, this study provides robust evidence of the impact of the policy on dental access for eligible children after 4 years of implementation, using a large nationally representative dataset from 2008 to 2018.

While monitoring the number of children accessing the CDBS is relevant and reliable, simply monitoring the number of children accessing the schedule is insufficient to assess its overall performance against the government's policy objectives (National Aboriginal Community Controlled Health Organisation [6, 32]). Future studies should investigate the impact of the scheme on improving other oral health outcomes, such as the reduction in untreated dental decay, unmet dental needs, or fewer dental hospitalisations.

## 5 Conclusion

This study examined how the CDBS policy improved dental attendance among children and adolescents from low‐income families, using data from the LSAC from 2008 to 2018. The empirical findings, from the conventional regression methods and the PSM methods, identified that there was a statistically significant increase in dental service use due to the introduction of the CDBS. The estimates indicated that the implementation of the CDBS was associated with a 6.4–6.7 percentage point increase in dental visits among eligible children.

## Ethics Statement

The ethical approval for the LSAC was from the Australian Institute of Family Studies Ethics Committee. There was no further need to get ethical approval as this study used an anonymised and unrestricted secondary dataset.

## Conflicts of Interest

The authors declare no conflicts of interest.

## Data Availability

The data that support the findings of this study are available from the Australian Institute of Family Studies. Restrictions apply to the availability of these data, which were used under license for this study. Data are available from https://dataverse.ada.edu.au/ with the permission of the Australian Institute of Family Studies.

## Appendix 1

### Placebo Test

We conducted a placebo implementation year and used the year 2012 as a placebo implementation year instead of the year 2014. Data were collected from 2008 to 2012. Table A1 presents the results.

**TABLE A1:** Difference‐in‐differences regression of placebo test.

Variables	Fixed‐effects panel analysis	Random effects panel analysis	Pooled data regression
	Coeff. (Robust SE)	Coeff. (Robust SE)	Coeff. (Robust SE)
DiD estimate (β_3_)	0.009 (0.019)	0.017 (0.017)	0.012 (0.019)
Control variables	Yes	Yes	Yes
Observations	12 713	12 713	12 713

*Note:* Robust standard errors are in the brackets. All regressions were clustered at the individual level. Fixed‐effect panel data and pooled data regressions were weighted. All regression models controlled for child, family, and residential characteristics.

The results showed no significant changes in dental visits of eligible children relative to non‐eligible children in the models in the DiD placebo regression, supporting our DiD design.

### Full Regression Results for DiD Models

**TABLE A2:** Difference‐in‐differences regression results.

Variable	Fixed‐effects panel data analysis		Random effects panel data analysis	Pooled data analysis
	Coeff. model 1	Coeff. model 2	Coeff. model 3	Coeff. model 4
Post (*β*_1_)	0.094*** (0.009)	−0.099*** (0.013)	−0.111*** (0.012)	−0.113*** (0.013)
Treat (*β*_2_)	−0.029** (0.012)	−0.009 (0.011)	−0.024** (0.010)	−0.024** (0.010)
**DiD estimate (*β***_**3**_ **)**	0.063*** (0.015)	0.064*** (0.015)	0.063*** (0.013)	0.061*** (0.014)
Child's characteristics				
Age		0.001*** (0.000)	0.002*** (0.000)	0.002*** (0.000)
Brushing teeth (< 2 times/day)		0.006 (0.009)	−0.032*** (0.007)	−0.053*** (0.008)
Ever had dental problems		0.309*** (0.012)	0.268*** (0.008)	0.260*** (0.009)
Gender (male)			−0.027*** (0.008)	−0.026*** (0.008)
Indigenous status (yes)			−0.084*** (0.023)	−0.061*** (0.024)
Family's characteristics				
Biological parents co‐habit (no)		−0.037* (0.019)	−0.024** (0.010)	−0.013 (0.011)
Education of mother (< bachelor)		−0.001 (0.018)	−0.004 (0.008)	0.005 (0.008)
Employment of mother (no)		−0.003 (0.011)	−0.025*** (0.008)	−0.029*** (0.009)
Health status of mother (fair/poor)		−0.001 (0.015)	−0.014 (0.012)	−0.007 (0.013)
Mother's smoking status (yes)		−0.013 (0.017)	−0.061*** (0.010)	−0.064*** (0.011)
Speaking English at home (no)		−0.166*** (0.047)	−0.079*** (0.012)	−0.066*** (0.013)
Homeowner (no)		0.022 (0.015)	−0.025*** (0.009)	−0.036*** (0.010)
Number of siblings		0.032*** (0.005)	0.011*** (0.003)	0.008** (0.003)
Family income (Highest: reference)				
Second quartile		0.026** (0.012)	0.002 (0.009)	−0.003 (0.010)
Third quartile		0.020 (0.014)	−0.024** (0.010)	−0.032*** (0.011)
Last quartile		0.015 (0.015)	−0.042*** (0.011)	−0.056*** (0.012)
SEIFA (Most advantaged area: reference)				
Second quartile		−0.010 (0.016)	−0.055*** (0.010)	−0.059*** (0.010)
Third quartile		−0.042** (0.018)	−0.104*** (0.010)	−0.117*** (0.011)
Last quartile		−0.048** (0.021)	−0.121*** (0.011)	−0.137*** (0.011)
Observation	22 985	22 984	22 984	22 984

*Note:* *, **, ***‐Statistically significant at 10%,5% and 1% level, respectively. Robust standard errors are in the brackets. Fixed‐effect panel data and pooled data regressions were weighted. All regressions were clustered at the individual level.

### Sensitivity Analysis

**TABLE A3:** Sensitivity analysis (without 2014 observations).

Variables	Fixed‐effects panel analysis	Fixed‐effects panel analysis
	Coeff. (Robust SE)	Coeff. (Robust SE)
DiD estimate (*β*_3_)	0.065*** (0.018)	0.061*** (0.018)
Control variables	No	Yes
Observations	19 221	19 220

*Note:* ***‐Statistically significant at1% level. Robust standard errors are in the brackets. Regressions were weighted and clustered at the individual level.

### Propensity Score Matching

Table A4 presents the results of the population‐weighted logit regression to assess the common support region.

**TABLE A4:** Logit model for Child Dental Benefits Schedule assignment.

Variable	Coeff. (Robust SE)
Gender (male)	0.068**(0.034)
Biological parent co‐habit (no)	0.677***(0.047)
No. of children in household	0.342***(0.018)
Mother's education (< bachelor)	0.245***(0.035)
Mother's employment (no)	2.793***(0.102)
Mother working * child’ age	−0.017***(0.000)
Mother's smoking (yes)	0.283***(0.049)
Mother's health (fair/poor)	0.194***(0.061)
Living in own house (no)	0.714***(0.043)
Household income (Highest: reference)	
Second quartile	1.419***(0.056)
Third quartile	2.311***(0.057)
Last quartile	2.006***(0.059)
SEIFA (Most advantaged area: reference)	
Second quartile	0.447***(0.051)
Third quartile	0.662***(0.051)
Last quartile	0.879***(0.051)
Observations	22 984

*Note:* ***‐Statistically significant level at1%; Mother working*child's age: We used transformation of variables (interaction term) in the model to control for the effect of the child's age while achieving the balance for covariates in the matching.

After identifying the overlap region, we matched the propensity scores within the region of common support. Observations in the unsupported region were dropped, and the final sample consisted of 3184 treated and 19,539 untreated observations. The overlap region is presented in Figure A1.

Table A5 presents the covariate balancing test in the matching methods, showing that the standardised difference of means of all covariates decreased after matching.

**TABLE A5:** Balance of baseline covariates across treatment and comparison groups before and after matching.

Variable	Standardised mean differences (%)			
	Unmatched	PSM	NMM	IPW
		Matched	Matched	Weighted
Gender (male)	6.68	3.97	1.01	−1.75
Biological parent, not co‐habits	60.94	−1.84	0.63	2.07
No. of children in household	25.98	3.58	7.14	−0.79
Mother's employment (no)	19.42	15.41	0.81	−11.59
Mother working*child's age	45.54	11.29	6.40	−16.82
Mother's education (< bachelor)	30.08	−0.13	1.26	−1.54
Mother's smoking (yes)	18.50	6.37	0.85	5.89
Mother's health (fair/poor)	22.93	9.61	0.27	−0.60
Living in own house (no)	39.50	8.59	1.35	7.45
Household income (Highest: reference)				
Second quartile	−27.35	0.69	0.35	0.57
Third quartile	44.64	−0.89	0.57	−1.16
Last quartile	44.12	0.64	−0.64	0.48
SEIFA (Most advantaged area: reference)				
Second quartile	−10.68	−2.88	0.08	−2.46
Third quartile	7.53	0.49	0.21	−1.05
Last quartile	34.66	1.81	−0.45	3.11

Table A6 presents the variance ratios of the covariates between the treated and untreated groups before and after matching.

**TABLE A6:** Variance ratios before and after matching.

Variable	Unmatched	Variance ratios		
		PSM	NMM	IPW
		Matched	Matched	Weighted
Gender (male)	0.99	0.99	1.00	1.00
Biological parent, not co‐habits	1.79	1.00	1.00	1.01
No. of children in the household	1.56	1.11	1.27	1.07
Mother's employment (no)	1.21	1.15	1.01	0.93
Mother working*child's age	2.77	1.00	1.15	0.78
Mother's education (< bachelor)	1.04	1.00	1.00	1.00
Mother's smoking (yes)	1.37	1.10	1.01	1.09
Mother's health (fair/poor)	1.80	1.24	1.01	0.99
Living in own house (no)	1.42	1.05	1.01	1.04
*Household income* (Highest: reference)				
Second quartile	0.67	1.01	1.01	1.01
Third quartile	1.46	1.00	1.00	1.00
Last quartile	1.45	1.00	1.00	1.00
SEIFA (Most advantaged area: reference)				
Second quartile	0.88	0.96	1.00	0.97
Third quartile	1.08	1.00	1.00	0.99
Last quartile	1.35	1.01	1.00	1.02
